# Late arthroscopic retrieval of a bullet from hip joint

**DOI:** 10.4103/0019-5413.54764

**Published:** 2009

**Authors:** Ravi K Gupta, Varun Aggarwal

**Affiliations:** Department of Orthopaedics, Government Medical College Hospital, Chandigarh-160 047, India

**Keywords:** Arthroscopy, bullet injury, hip

## Abstract

We describe a case of arthroscopic retrieval of a bullet from the hip joint of an 18-year-old boy, who sustained the injury four months back, accidentally, while bird hunting with a country made shotgun. The surgery was performed with the standard ordinary instrumentation of knee arthroscopy. The patient became pain-free the same evening and started partial weight bearing on the next day of surgery. At 13 months follow-up, the patient had returned to normal activity without any functional limitations.

## INTRODUCTION

The seven reports of arthroscopic-assisted retrieval of bullets within few days of injury from the hip joints have been reported in the English language literature.^1^–^7^ Out of which only three have been retrieved purely by arthroscopy.^1^,^5^,^6^ In the other four reports,^2^–^4^,^7^ a limited open approach has been used around the bullet tract or up to the capsule of the hip joint, which was then penetrated with arthroscopic trocar.

We describe a case report of late retrieval of a bullet from the hip joint, arthroscopically. The procedure was performed with traditional arthroscopic instrumentation without the use of a dedicated canulated hip arthroscopy instrumentation set. We herewith present the technical difficulties related to the late retrieval of the projectile from the hip joint.

## CASE REPORT

**Figure 1 (a, b):**
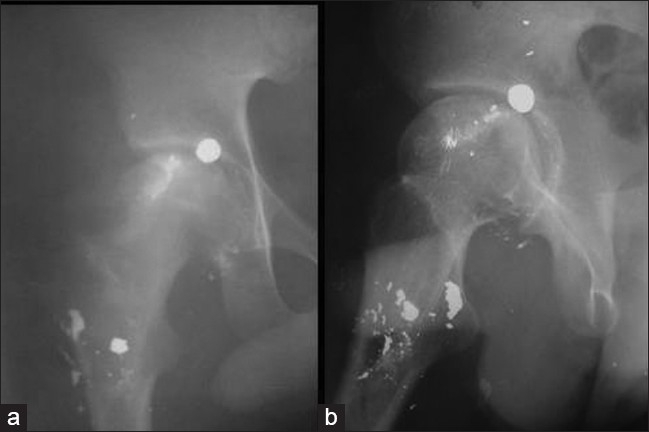
X-ray (anteroposterior and lateral views) of hip joint showing bullet in the acetabulum

An 18-year-old male student sustained an accidental gun shot injury to the left hip region, with a country made lead shotgun, during bird hunting. There were multiple small entry wounds in the left trochanteric area caused by tiny pellets. There was no fracture. Initially he was given local wound care and systemic antibiotics. After the wounds healed, the patient was unable to bear the weight on the left hip joint due to pain and started non-weight bearing walking with crutches. He continuously complained of a sudden severe catching pain in the hip joint in a particular position of the thigh while swaying the limb during the crutch walking, and in the bed during change of posture. He was taking nonsteroidal anti-inflammatory drugs and massage for the same. He reported four months after injury. His plain X-rays (AP and lateral view) of hip joint showed multiple pellets in the region of the proximal femur with one pellet lodged in the hip joint [Figure 1a and b].

**Figure 2:**
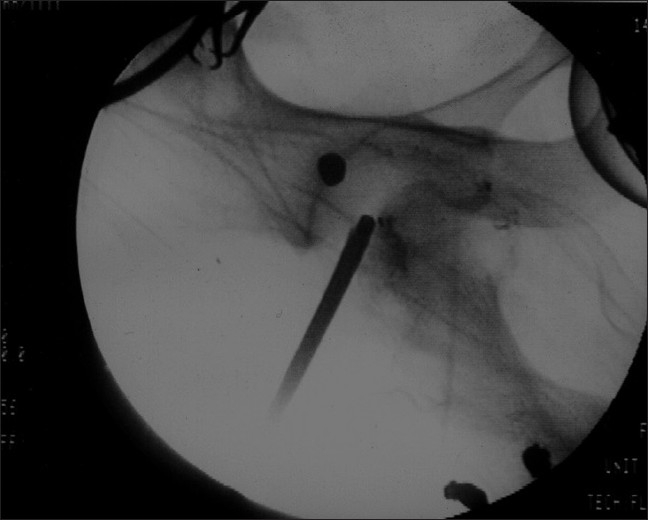
Image guided location of arthroscopic trocar in the vicinity of bullet

The patient was taken up for an arthroscopic retrieval of the bullet with a preparedness of an arthrotomy in case of failure. The patient was positioned supine on the fracture table with a standard mid-line perineal post. In spite of the thin built of the patient, distraction at the hip joint was difficult and required heavier traction. The standard antero-lateral portal and the lateral (supra trochanteric and anterior partrochanteric) portals were used. A 30° scope was used. In the absence of the arthroscopic pump, the effect of gravity was used in distending the hip joint by raising the height of the irrigation fluid. The visualization of the bullet was initially not easily possible due to chronicity having resulted in the growth of organized scar tissue around the projectile. Debridement of the hyper-trophic synovium was done with the shaver, resulting in some bleeding inside the joint, making the arthroscopic picture blurred. The scope was placed adjacent to the bullet with the help of the image intensifier [Figure 2]. Visualization was possible by lowering the blood pressure of the patient, and raising the fluid pressure inside the hip joint by enhancing the height of the fluid bottles and lowering the operation table. The surrounding area was cleared with the shaver blade and thermal ablator leading to visualization of the bullet, which was lying firmly impacted in the supero-lateral acetabular wall.

**Figure 3:**
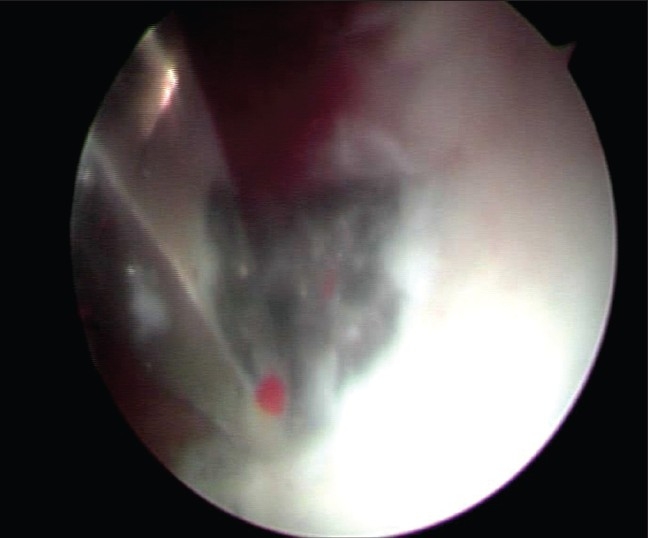
Disimpaction of bullet with a fine osteotome

**Figure 4:**
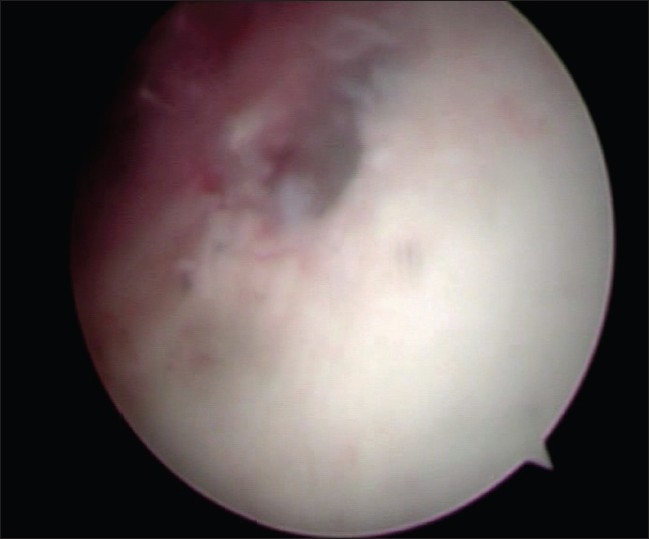
Crater in the acetabular wall

**Figure 5:**
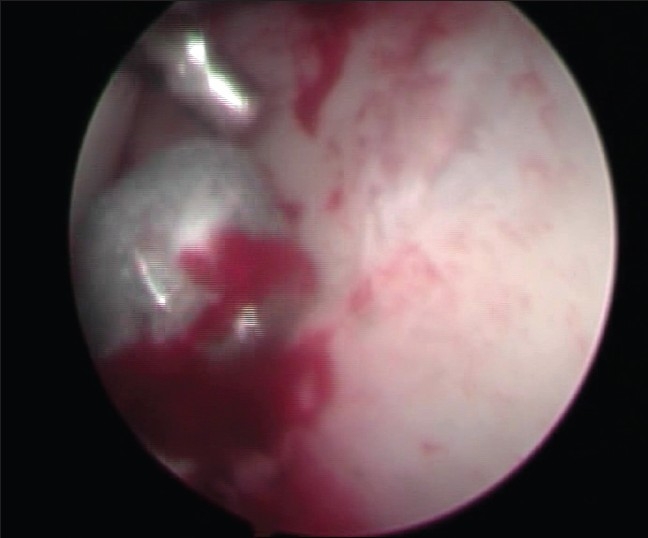
Manipulation of bullet with a hook probe

It was not possible to move the bullet with the probe or the canula of the arthroscopic trocar. A long thin osteotome from the revision hip arthroplasty set was used to disimpact the bullet [Figure 3]. The disimpaction of the bullet left a well circumscribed crater in the acetabular wall [Figure 4]. The bullet was manipulated with the probe to a convenient location in the hip [Figure 5], where grasping was possible with a loose body grasper.

**Figure 6:**
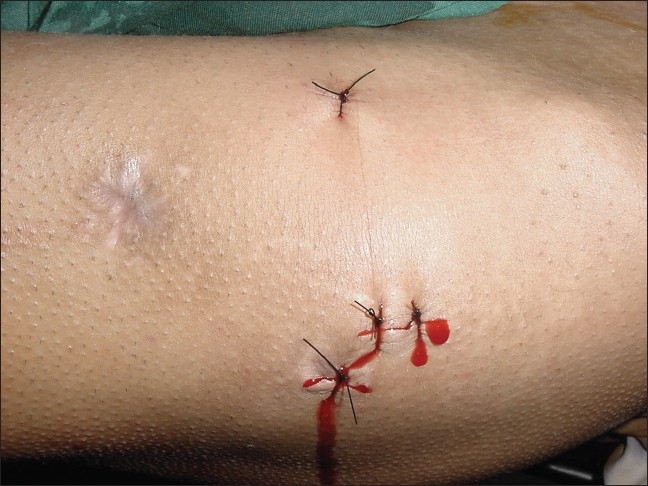
Clinical photograph showing sutured portals

**Figure 7:**
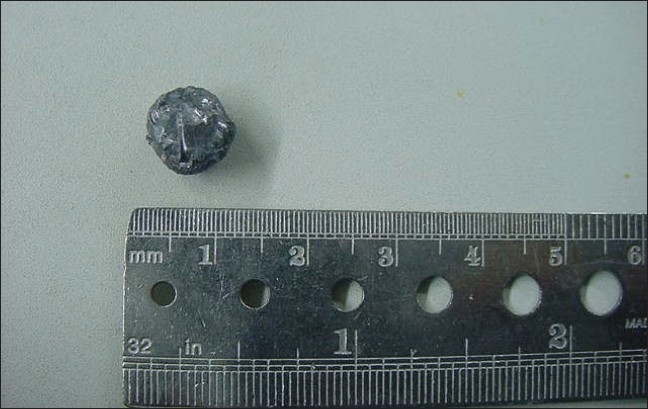
Retrieved bullet

Arthroscopic examination of periphery of the joint was made with 30° and 70° scopes to rule out any other loose fragments. No major defect in the femoral head was noticed except for a small abrasion in the supero-posterior area. The total duration of the procedure was 70 minutes. The three portals used in the procedure were sutured [Figure 6]. The removed bullet was roughly spherical shaped with approximate one cm diameter [Figure 7].

The patient became pain free the same evening and started partial weight bearing on the next day of surgery. However, he complained of mild temporary numbness in the perineal region probably resulting from heavier traction, which recovered completely in three months. At one month, the patient started normal full weight bearing. The hip flexion on the affected side was short by 30° than the normal, abduction and adduction were short by 20° and five degrees respectively and external and internal rotations by five and 10° respectively. At 13 months follow-up, the patient had returned to normal activity without any functional limitations.

## DISCUSSION

Hip being a deep joint surrounded by heavy muscles all around, the open access to the joint is a major procedure requiring more dissection, more blood loss and delayed post operative rehabilitation. A danger of damage to the blood supply of the femoral head is an additional serious risk factor especially with the posterior approach. Although in the absence of the dedicated hip arthroscopy instrumentation with long handles, the chances of failure of the procedure are high, making an attempt with the existing technology is worthwhile to reduce the morbidity of the open procedure.

During the procedure, the distraction of the hip joint required more traction than we normally used in hip arthroscopy. This may be due to the formation of scar tissue around the projectile resulting from a delay of four months. However, the patient being of a thin built with less muscles, we were able to overcome this, although it resulted in temporary neuropraxia in the perineal region due to pressure of the perineal post.

We used a thin long curved osteotome for disimpacting the projectile from the acetabulum. Before introducing the osteotome, we had already unsuccessfully tried moving the bullet with a probe and a blunt arthroscopic trocar repeatedly. Before abandoning the procedure and changing over to open arthrotomy, the attempt of using the osteotome proved fertile. However, we were very cautious during the introduction of the osteotome and used fluoroscopic navigation to avoid slipping of the osteotome. After the disimpaction, we used a probe hook to manipulate the disimpacted bullet towards the convenient location in the joint where the grasper could be easily introduced. The thin built of the patient further facilitated the reach of our instruments into the hip joint.

The compelling reason of surgical extraction of an intraarticular bullet in the present case was excruciating pain suffered by the patient and his inability to lead a normal life. The location of the bullet in the hip joint resulting in catching of the bullet in certain positions of the thigh had made the patient's life miserable. Even otherwise, the extraction of the bullet from the hip joint has been indicated to avoid the long term risk of mechanical arthritis, and systemic lead toxicity caused due to dissolution and absorption of the lead through the synovial fluid.^4^,^8^–^10^ The risk of being a potential nidus of infection, although mentioned in literature,^4^,^11^ was probably negligible in our case at four months post-injury.

We have not used a pre-operative CT scan in the present case. However, the two standard antero-posterior and lateral views of the preoperative X rays and the visualization of the joint through a complete arch of rotation of C arm, per operatively, helped us to rule out the other intraarticular pathologies even in the medial articular space where the arthroscopic visualization is difficult.^3^ Although we agree that the availability of the facility of CT scan pre-operatively is an additional advantage, for more accurate planning and better pre-operative documentation, absence of the facility should not be a limiting factor for undertaking the surgery.

With successful arthroscopic retrieval, we could avoid several disadvantages of an open procedure including significantly diminished blood loss, cosmetic incisions, decreased risk of osteonecrosis of the femoral head and shortened recovery time.^6^,^7^ All the potential complications of the hip arthroscopy were discussed with the patient before undertaking the procedure. There is a report of cardiac arrest resulting from extravasation of the irrigation fluid through the fracture line into the abdomen during the arthroscopic removal of a loose body from the hip joint of a patient with an acetabular fracture.^12^ A theoretic risk of developing the same complication by introduction of the fluid through the bullet tract has also been expressed.^4^ In the present case, however, having no fracture line, and being undertaken at a delay of four months, resulting in complete healing of the bullet tract, this risk was negligible. Other risks to be kept in mind include perineal numbness/necrosis related to the traction post, radiation exposure and injury to the femoral neurovascular bundle anteriorly and sciatic nerve posteriorly.

We feel that a small residual crater in the weight-bearing area of the acetabulum left after the extraction of the bullet, in the present case, is unlikely to cause immediate problem because the large sized spherical femoral head will be able to walk over it without catching. However, the long term possibility of a secondary osteoarthritis is a risk to be kept in the mind and we have advised the patient to be on regular follow-up at least once in six months. In conclusion, we feel that it is worthwhile to attempt for arthroscopic retrieval of foreign bodies from the hip joint even in the absence of specialized equipment. This reduces the morbidity of the patient.
